# Sedative effects of propofol and risk factors for excessive sedation in the endoscopic treatment of biliary and pancreatic diseases

**DOI:** 10.1002/deo2.417

**Published:** 2024-09-02

**Authors:** Yuta Maruki, Susumu Hijioka, Shin Yagi, Tetsuro Takasaki, Mark Chatto, Soma Fukuda, Daiki Yamashige, Kouhei Okamoto, Daiki Agarie, Hidenobu Hara, Yuya Hagiwara, Yoshikuni Nagashio, Chigusa Morizane, Miyuki Sone, Takuji Okusaka, Yutaka Saito

**Affiliations:** ^1^ Department of Hepatobiliary and Pancreatic Oncology National Cancer Center Hospital Tokyo Japan; ^2^ Molecular Oncology The Jikei University School of Medicine Tokyo Japan; ^3^ Department of Gastroenterology Makati Medical Center Makati City Philippines; ^4^ Department of Diagnostic Radiology National Cancer Center Hospital Tokyo Japan; ^5^ Endoscopy Division National Cancer Center Hospital Tokyo Japan

**Keywords:** biliary and pancreatic diseases, endoscopic cholangiopancreatography, endoscopic ultrasound‐guided intervention, excessive sedation, propofol

## Abstract

**Objectives:**

The safety and effectiveness of propofol in more complex endoscopic procedures, such as endoscopic retrograde cholangiopancreatography, remain unknown. Thus, we aimed to evaluate propofol sedation during endoscopic cholangiopancreatography, ultrasound‐guided intervention, and gastroduodenal stenting and examine risk factors for excessive sedation.

**Methods:**

We retrospectively analyzed data from 870 patients who underwent endoscopic treatment with propofol sedation for biliary and pancreatic disease between October 2020 and September 2021. Sedation included propofol and fentanyl, with continuous monitoring of vital signs and the bispectral index. The assessed risk factors included age, complications, body mass index, treatment duration, and specialty.

**Results:**

Distal bile duct treatment (*n* = 367), hilar bile duct treatment (*n* = 197), post‐small‐intestinal reconstruction treatment (*n* = 75), endoscopic ultrasound‐guided intervention (*n* = 140), and gastrointestinal obstruction treatment (*n* = 91) were performed. The rates of excessive sedation, hypoxemia, and hypotension were 7.8%, 6.0%, and 1.8%, respectively. Post‐small‐intestinal reconstruction treatment had the highest incidence rate of excessive sedation (16%), whereas endoscopic ultrasound‐guided intervention had the lowest incidence rate (4.3%). Multivariate analysis revealed significant associations between excessive sedation and comorbid sleep apnea, obesity, and prolonged procedural time.

**Conclusions:**

Obesity, sleep apnea syndrome, and prolonged procedure time are risk factors for excessive sedation related to propofol use. Thus, sedation techniques should be tailored for these patients.

## INTRODUCTION

The American Society for Gastrointestinal Endoscopy (ASGE) guidelines emphasize that “it is the responsibility of the endoscopist to make every effort to ensure that endoscopy is consistent with the best interests and safety of the patient.”^1^ The procedure time for endoscopic retrograde cholangiopancreatography (ERCP) has become increasingly longer, and ERCP has become more invasive and common in older patients. Additionally, new techniques for biliary drainage, including endoscopic ultrasound‐guided intervention (EUS‐IV), have gained momentum. Sedation and analgesia play critical roles in facilitating the stable completion of these procedures.

The American Society for Gastrointestinal Endoscopy guidelines for therapeutic endoscopy,^1^ including ERCP, strongly recommend using opioids with benzodiazepines, including midazolam; however, the guidelines only suggest propofol‐based sedation. In contrast, the Japan Gastroenterological Endoscopy Society guideline^2^ strongly recommends sedation with propofol and midazolam. However, sedation with midazolam has been used in daily practice at several institutions. Propofol broadly inhibits the central nervous system and activates gamma‐aminobutyric acid receptors.^3^ It has a short onset and duration of action with a half‐life of 2–8 min. In contrast to midazolam, the half‐life of the blood concentration after discontinuing continuous intravenous infusion is not affected by the time of administration.

Since 2000, numerous prospective studies have been conducted internationally on propofol sedation for ERCP. Benzodiazepine (midazolam) was used as a control and was compared with propofol. These studies reported favorable arousal in the propofol group and emphasized the safety of its use in older patients.^4^, ^5^, ^6^ Recently, a Korean study comparing propofol and midazolam for nonanesthetic sedation in patients aged >80 years reported no difference in safety between the two groups.^7^ A meta‐analysis of advanced endoscopy, including ERCP and EUS, showed that sedation with propofol resulted in a shorter recovery time to wakefulness than that with benzodiazepines. This phenomenon was observed without significant differences in hypoxemia, hypotension, or procedure time.^8^ A combination of propofol and fentanyl is safe and reduces the amount of propofol required for ERCP.^9^ Therefore, fentanyl may be a safe alternative to propofol as an analgesic.

The Japan Gastroenterological  Endoscopy Society has published a second edition of its sedation guidelines^2^ for gastrointestinal endoscopy in Japan. This edition strongly recommends using propofol as a sedative in endoscopic procedures. However, because of insurance coverage issues and concerns regarding propofol safety when administered by non‐anesthesiologists, sedation with propofol has not become standard practice. Ogawa et al. investigated whether propofol sedation could be safely performed in older patients (>70 years) during ERCP and reported incidence rates of hypoxemia and hypotension ranging from 3.1% to 4.9% and 2.3% to 6.3%, respectively.^10^ However, reports on propofol use in ERCP are scarce, and no previous reports exist on EUS‐IV or ERCP use in the reconstructed intestinal tract. Thus, this study aimed to assess the safety of propofol in treating biliary and pancreatic diseases in Japanese patients. It specifically evaluated propofol sedation during biliopancreatic endoscopic procedures at an institution and conducted a detailed analysis that included identifying risk factors for excessive sedation.

## MATERIALS AND METHODS

### Study design and objectives

This was a single‐center, retrospective analysis of patients who underwent cholangiopancreatic endoscopic procedures using propofol as a sedative between October 2020 and September 2021. Patients who underwent cholangiopancreatoscopy without propofol for reasons such as pediatric treatment or allergies were excluded from this study.

Cholangiopancreatic endoscopic treatment included ERCP, EUS‐IV, endoscopic retrograde cholangiography for bowel reconstruction, and gastrointestinal endoscopy for duodenal obstruction.

The hospital's Institutional Review Board (IRB) approved this study (IRB No. 2018–149). The IRB did not require informed consent.

## METHODS

Sedation was induced using propofol, and fentanyl was used as an analgesic. An initial bolus dose of 0.5 mg/kg was administered for propofol, followed by a maintenance dose of 1.5 and 2.0 mg/kg/h. Additional doses were administered when necessary to increase sedation levels. Regarding fentanyl usage, an initial dose of 0.5 µg/kg was administered, with an extra single dose of 0.2 µg/kg administered if inadequate analgesia was suspected. Numerous body movements during the procedure were considered insufficient analgesia.

All patients were monitored by assessing their bispectral index (BIS), respiratory rate, oxygen saturation (SpO_2_), periodic blood pressure (every 2.5 min), heart rate, and three‐lead electrocardiogram results. The respiratory rate was continuously and noninvasively monitored using the Masimo Rainbow Acoustic Monitoring system (Masimo Rainbow Acoustic Monitoring; MASIMO Japan). If the respiratory rate was <10 breaths/min, additional fentanyl was prohibited. BIS was measured using a forehead‐attached BIS sensor connected to a monitor, with the targeted depth of sedation set between 60 and 70 based on BIS monitoring (BIS Monitoring System, Covidien Japan Ltd.).

At the start of all procedures, all patients received 2 L/min of oxygen through a nasal cannula, and oral suctioning was performed when necessary. Additionally, all patients were started in the supine position.

One attending physician, a non‐anesthesiologist, was designated as the dedicated sedation physician responsible for adjusting propofol dosage during the endoscopic procedure.

### Outcomes

The primary endpoints included the incidence rate of excessive sedation, associated risk factors, and rate of excessive sedation categorized according to the procedure. Excessive sedation was considered when a patient experienced fluctuations in vital signs during the procedure, requiring actions such as endoscope removal, hypotension requiring additional fluid administration, norepinephrine, and other blood pressure medications, and further airway management.

This study examined several risk factors for excessive sedation, including age, body mass index (BMI), treatment for malignancy, albumin level, C‐reactive protein level, sleep apnea syndrome (SAS), chronic obstructive pulmonary disease, and history of heart disease. It also assessed the frequency of oversedation across different examination categories, including distal bile duct treatment, hilar bile duct treatment, post‐small intestinal reconstruction treatment, EUS‐IV, and treatment of gastrointestinal tract obstruction. The duration of the endoscopic procedure was categorized as prolonged if it lasted ≥70 min.

In this study, hypotension and hypoxemia during endoscopic procedures were defined as excessive sedation; hypoxemia was defined as a decrease in SpO_2_ to <85% for >15 s, and hypotension was defined as systolic blood pressure <80 mmHg for >5 min (3 consecutive measurements at 2.5‐min intervals).

The severity of excessive sedation was determined according to the Cotton criteria^11^ and modified as follows:

For decreased SpO_2_, supplemental oxygen and sedation were classified as mild, airway clearance and procedure discontinuation requiring endoscope removal as moderate, intensive care unit (ICU) admission as severe, and death as fatal.

For hypotension, additional fluid and sedation were considered mild, the use of antihypertensive medications was moderate, ICU admission was severe, and death was fatal.

### Statistical analyses

Correlations between risk factors in cases of excessive sedation were analyzed using Fisher's exact test and logistic regression. The cutoff value for the risk factors was set at a clinically significant point near the value, with maximum sensitivity and specificity (the Youden index) derived from the receiver operating characteristic curve. All tests were two‐tailed, and statistical significance was set at *p* < 0.05. Statistical analyses were performed using IBM SPSS Statistics for Windows, version 22.0 (IBM Corp.).

## RESULTS

**FIGURE 1:**
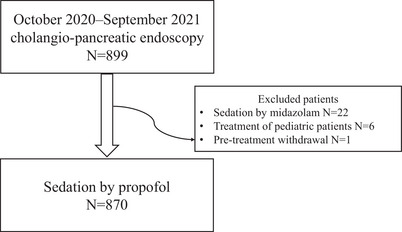
Chart showing the number of biliopancreatic endoscopy cases during the study period and the participants analyzed in this study who were sedated with propofol.

This study included 899 patients, among whom 870 were sedated with propofol (Figure 1). Pediatric patients (n = 6) were excluded from the analysis because they received ketamine for sedation. If patients were apneic before initiating sedation, the procedure was stopped, and the patients were excluded from the study. Baseline patient characteristics are presented in Table 1. Treatment for malignancy accounted for 96% of patients, including 39% and 34% with low albumin (<3.0 g/dL) and high C‐reactive protein (>5.0 mg/L) levels, respectively. Table 2 presents details of the treatments; 22% of the treatments lasted >70 min.

**TABLE 1:** Patient characteristics at the time of treatment.

Characteristics	*N* (%) (*N* = 870)
Sex	
Male	522 (60)
Female	348 (40)
Median age (range)	63 (17–93)
≥75 years	227 (26)
<74 years	643 (74)
Benign or malignant diseases	
Benign	32 (4)
Malignant	738 (96)
Median body mass index score (range)	20.14 (13.75–39.36)
≥25 kg/m^2^	105 (12)
<25 kg/m^2^	765 (88)
Median albumin level (range)	3.2 (1.1–5.0)
≥3.0 g/dL	528 (61)
<3.0 g/dL	342 (39)
Median C‐reactive protein level (range)	2.59 (0.01–43.07)
≥3.0 mg/L	412 (47)
<3.0 mg/L	458 (53)
Median creatinine score (range)	0.72 (0.22–6.72)
≥1.0 mg/dL	113 (13)
<1.0 mg/dL	757 (87)
Comorbidity	
Sleepless apnea syndrome	9 (1)
Chronic obstructive pulmonary disease	9 (1)
Cardiovascular disease	40 (5)

**TABLE 2:** Details of treatment.

Procedure details	*N* (%) (*N* = 870)
Median procedure time (range)	40 min (6–220)
≥70 min	191 (22)
<69 min	679 (78)
Median propofol accumulation (range)	250 mg (26–1787)
≥400 mg	178 (20)
<399 mg	692 (80)
Median fentanyl accumulation (range)	0.03 mg (0–0.30)
≥0.1 mg	63 (7)
<0.1 mg	807 (93)
Procedure category	
Distal bile duct treatment	367 (42)
Hilar bile duct treatment	197 (23)
Post‐small intestinal reconstruction treatment	75 (9)
EUS intervention	140 (16)
Treatment of gastrointestinal obstruction	90 (10)

Transpapillary treatment was performed in 65% of patients, and 34 (45%) of the 75 patients with reconstructed bowel were treated using a double‐balloon endoscope. Additionally, 140 patients who underwent EUS‐IV were included as follows: 76, 27, 16, and 21 patients for EUS‐guided hepaticogastrostomy, EUS‐guided choledochoduodenostomy, EUS‐guided cyst drainage, and other EUS‐IV, respectively.

The rate of excessive sedation, the study's primary outcome, was 7.8% (*n* = 68). Hypoxemia was observed in 6% of patients (*n* = 53). Details of excessive sedation are summarized in Table 3. Ten (19%) patients with hypoxemia had moderate or severe disease, and only mild hypotension was reported. One patient with hypoxemia experienced severe symptoms and was admitted to the ICU. The patient had serious adverse events owing to air embolus hypoxemia and was admitted to the ICU because of decreased consciousness and other symptoms. Table 4 summarizes the 10 cases of moderate or severe hypoxemia. One patient underwent an endoscopic papillectomy for a 30‐mm stalked adenoma. The patient could resume treatment after inserting a nasal tube to secure the airway. Additionally, no excessive sedation was observed during EUS‐IV in patients with moderate or severe hypoxemia.

**TABLE 3:** Details and severity of excessive sedation cases.

Details of excessive sedation	*N* (%)
Excessive sedation	68 (7.8)
Hypoxemia	53 (6.0)
Hypotension	15 (1.8)

	Severity grade			
	Mild	Moderate	Severe	Fatal
Hypoxemia	43 (81)	9 (17)	1 (2)	0 (0)
(*N* = 53)				
Hypotension	15 (100)	0 (0)	0 (0)	0 (0)
(*N* = 15)				

**TABLE 4:** Details of cases of severe excessive sedation.

No.	Sex	Age (years)	Disease	Severity	Procedure category	BMI	Comorbidity	Propofol accumulation (mg)	Procedure time (min)	Additional treatment
1	F	51	Benign	Moderate	Distal	30.36	Cardiovascular disease	770	120	Nasotube insertion
2	M	51	Malignant	Moderate	Distal	28.32	None	560	130	Treatment interruption
3	F	67	Malignant	Moderate	Distal	17.56	None	339	50	Nasotube insertion
4	F	50	Malignant	Moderate	Gastrointestinal obstruction	27.51	None	135	30	Nasotube insertion
5	F	73	Malignant	Moderate	Distal	31.93	None	500	47	Nasotube insertion
6	M	72	Malignant	Severe	Post‐small intestinal reconstruction	17.3	None	333	90	ICU admission
7	F	66	Malignant	Moderate	Post‐small intestinal reconstruction	19.64	None	333	78	Nasotube insertion
8	M	79	Malignant	Moderate	Hilar	19.72	None	418	135	Nasotube insertion
9	M	78	Malignant	Moderate	Hilar	32.19	None	278	56	Nasotube insertion
10	M	59	Malignant	Moderate	Hilar	26.91	None	265	30	Nasotube insertion

Abbreviations: BMI, body mass index; F, female; ICU, intensive care unit; M, male.

The risk factors for excessive sedation were analyzed based on patient background and procedures. In the multivariate analysis, the following three factors showed significant associations with excessive sedation: high BMI (≥25 kg/m^2^), presence of SAS, and prolonged procedure time (>70 min; Table 5).

**TABLE 5:** Risk factors in cases of excessive sedation.

	Excessive sedation (*N*)			Univariate analysis		Multivariate analysis		
	+	‐	OR	95% CI	*p‐*value	OR	95% CI	*p‐*value
Age (years)								
≥75	21	206	1.29	0.77–2.04	0.39			
<74	47	596						
BMI								
≥25 kg/m^2^	19	86	3.23	1.72–4.53	<0.001	2.88	1.53–5.20	<0.001
<25 kg/m^2^	49	716						
Albumin level								
≥3.0 g/dL	43	485	1.12	0.70–1.43	0.70			
<3.0 g/dL	25	317						
CRP								
≥3.0 mg/dL	36	376	1.28	0.79–2.01	0.38			
<3.0 mg/dL	32	426						
Creatinine								
≥1.0 mg/dL	14	99	1.83	0.91‐3.51	0.06			
<1.0 mg/dL	54	703						
SAS								
Yes	4	5	9.96	2.47–10.7	0.003	5.42	1.17–23.5	0.02
No	64	797						
COPD								
Yes	1	8	1.48	0.25–5.77	0.52			
No	67	794						
Cardiovascular disease								
Yes	2	38	0.62	0.17–2.16	0.76			
No	65	765						
Procedure time (min)								
≥70 min	26	165	2.39	1.41–3.97	0.002	2.39	1.39–4.04	0.001
<69 min	42	637						
Propofol accumulation								
≥400 mg	17	161	1.33	0.77–2.15	0.35			
<399 mg	51	641						
Fentanyl accumulation								
≥0.1 mg	8	55	1.80	0.71‐4.06	0.14			
<0.1 mg	60	747			
Post‐small intestinal reconstruction								
Yes	12	63	2.23	1.16–4.30	0.03	1.94	0.88–4.02	0.08
No	62	727						

Abbreviations: BMI, body mass index; CI, confidence interval; COPD, chronic obstructive pulmonary disease; CRP, C‐reactive protein; OR, odds ratio; SAS, sleep apnea syndrome.

**FIGURE 2:**
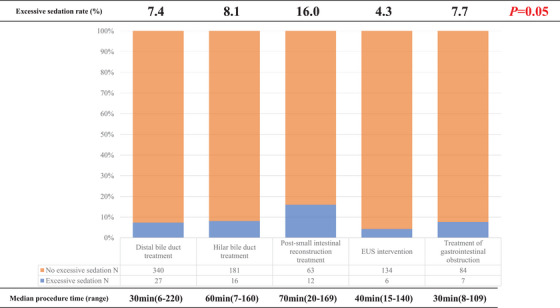
Chart depicting the percentage of excessive sedation according to examination category.

The incidence rates of excessive sedation varied according to the procedure category, with the highest and lowest rates observed for reconstructed bowel treatment (16%) and EUS‐IV (4.3%), respectively (Figure 2). There was a tendency for higher risks of excessive sedation during reconstructed bowel treatment.

## DISCUSSION

This study assessed the safety of propofol sedation administered by non‐anesthesiologists during therapeutic endoscopy, specifically ERCP and EUS‐IV, which are considered advanced procedures. The study focused on the incidence of excessive sedation and risk factors associated with this outcome. The overall rate of excessive sedation in this study was 7.8%, with hypoxemia and hypotension occurring in 6% and 1.8% of patients, respectively. Hypotension was of mild severity, whereas 10 cases of hypoxemia were classified as moderate or severe. This is consistent with the findings of Ogawa et al., who reported 3.1%–4.9% and 2.3%–6.3% incidence rates of hypoxemia and hypotension, respectively, during non‐anesthesiologist‐administered propofol sedation for ERCP in older patients (age ≥70 years).^10^ Gotoda et al. reported cases of hypoxemia and a 1.2%–2.5% incidence rate of hypotension during propofol sedation for gastric ESD in older patients (age ≥70 years).^12^ Regarding excessive sedation, they reported incidence rates of 2.3%–5.0% and 1.2%–2.5% for hypoxemia and hypotension, respectively. Our study results indicated a higher incidence rate of excessive sedation associated with propofol than previously reported gastric ESD procedures. One reason for this discrepancy is the difference in positioning between the procedure and gastric ESD. The thorax and abdomen experience mechanical compression during ERCP in the supine position, resulting in restricted thoracic motion. This restriction can lead to decreased lung compliance, resulting in carbon dioxide (CO_2_) accumulation owing to hypoventilation and the potential development of atelectasis. Studies in young adults have measured changes in respiratory function in different positions (sitting, standing, and supine) and have shown that parameters, such as forced expiratory volume in 1 s, ratio of forced expiratory volume in 1 s to forced vital capacity, and percent lung capacity are lower in the supine position than in the sitting and standing positions.^13^ Because ERCP performed in the supine position results in limited pulmonary ventilation, prompt repositioning to left lateral recumbency or other measures should be considered if hypoxemic oversedation occurs.

Another essential feature of this study was that 96% of the included patients had malignancies. The most common reason for ERCP is biliary stone disease, which is a significant cause of hospitalization worldwide, with a prevalence of 3.2%–15.6% in Asia.^14^ Previous reports on sedation for ERCP have also shown that most target diseases are benign, with malignant disease accounting for 10%–30% of cases.^15^ Many of the patients in this study had malignant tumors, and the study demonstrated that propofol could be safely used during ERCP in patients whose general condition is unstable due to the presence of cachexia or other factors.

This study examined risk factors for excessive sedation associated with propofol use and identified high BMI (≥25 kg/m^2^), prolonged treatment (>70 min), and history of SAS as independent risk factors. These findings suggest that patients with obesity, high BMI, and a history of SAS face challenges in securing their airways. Benzodiazepine sedation induces deep sedation with respiratory depression in 85% of patients undergoing ERCP.^16^ Therefore, it is critical to consider factors that place patients at risk of respiratory depression during ERCP before the procedure. Additionally, Wani et al. reported that obesity was a risk factor for sedation‐related adverse events, particularly hypoxemia, associated with propofol use in advanced endoscopic procedures, including ERCP and EUS.^17^ A longer procedure time is also associated with increased risks of excessive sedation. This study included all endoscopic treatments for biliary and pancreatic diseases, and treatment difficulty and complexity varied across cases. In such a heterogeneous population, procedures lasting >1 h may be more invasive, potentially leading to increased oral secretions, making it more challenging to secure the airway. Therefore, highly invasive and prolonged procedures are associated with higher risks of excessive sedation. The European Society of Anaesthesiology and European Board of Anaesthesiology guidelines^18^ list heart disease, obstructive sleep apnea, severe obesity, renal failure, liver failure, and advanced age as risk factors for sedation. Patients with obstructive SAS are more susceptible to drug‐induced cardiopulmonary depression during deep sedation,^19^ underscoring the importance of recognizing obstructive SAS complications during endoscopic sedation. Furthermore, some patients may have undiagnosed obstructive SAS. Therefore, it is vital to ask about snoring and sleep apnea to identify potential risk factors for sedation. This study evaluated propofol sedation for the endoscopic treatment of biliary and pancreatic diseases without anesthesiologist assistance, considering the shortage of anesthesiologists in Japan. To reduce hypoxemia incidence, it is suggested that anesthesiologists perform the procedure for high‐risk patients. The use of propofol as a sedative agent under the supervision of an anesthesiologist will be considered for the endoscopic treatment of biliary and pancreatic diseases where prolonged procedures are anticipated or where a history of SAS or obesity is present, which were the three risk factors for excessive sedation identified in this study.

In our study, eight of the 10 patients with moderate‐to‐severe hypoxemia had a suction tube for aspirating sputum inserted into the nasal cavity and oxygen connected directly to the tube. This method provides a high flow of oxygen that cannot be delivered by the conventional nasal cannula method. Ishi et al. reported that administering oxygen by inserting a suction tube into the nasal cavity could provide a high oxygen flow and reduce moderate or severe hypoxemia during the procedure.^20^ A system that can appropriately manage cases with risks such as obesity or a history of SAS by inserting a suction tube into the nasal cavity could reduce the risk of severe excessive sedation.

One patient with hypoxemia experienced severe symptoms and was admitted to the ICU. The patient had biliary obstruction because of recurrent liver metastases in the reconstructed intestinal tract and required drainage tube exchange. Hypoxemia occurred during the procedure because of prolonged procedure time and the development of air embolization associated with CO_2_ insufflation. In double‐balloon endoscopy, insufflated CO_2_ fills the closed space, allowing air to enter the vessels and potentially causing air embolism. Two cases of air embolism have been reported during biliary stone clearance using balloon endoscopy.^21^, ^22^ In these cases, hypoxemia is unlikely to be influenced by propofol.

This study also evaluated the incidence of excessive sedation according to the endoscopic procedure category and found that EUS‐IV had the lowest incidence rate (4.3%), although not significantly different. Recently, EUS‐IV has become popular as a salvage procedure for transpapillary bile duct drainage. However, its technical difficulties and serious complications limit the number of centers that perform this procedure. Sedation during EUS‐IV is essential for ensuring a stable procedure. Ogura et al. retrospectively compared adverse events associated with EUS‐IV in older and non‐older patients and reported sedation‐related adverse events. They reported a significant reduction in hypoxemia in older patients using midazolam as a sedative and waveform capnography, with 6.4% of the older group having hypoxemia.^23^ We present data on the incidence of excessive sedation during EUS‐IV using propofol as a sedative. It is crucial to consider the appropriate sedation method for EUS‐IV.

This study had some limitations. As this was a single‐center, retrospective study, it may be limited because the assessment of outcomes per patient was not as standardized as it would have been in a prospective study where inter‐rater variability was more controlled. Therefore, a prospective study is required to collect data in a more defined manner. Additionally, this study did not assess postoperative alertness. An advantage of propofol sedation is its short wakefulness time, which has been reported in several studies. We expect that endoscopic treatment of biliary and pancreatic diseases will lead to reasonable levels of wakefulness, as observed in previous reports. However, further studies are required to determine whether similarly good arousal can be achieved during highly invasive procedures and with high‐dose propofol use.

## CONFLICT OF INTEREST STATEMENT

None.

## ETHICS STATEMENT

This study was approved by the Ethics Committee of the National Cancer Center Hospital (approval number: 2018–149). Obtaining informed consent, clinical trial registration/ registry numbers, and animal studies are N/A.
